# Balancing Theory and Practice in Respondent-Driven Sampling: A Case Study of Innovations Developed to Overcome Recruitment Challenges

**DOI:** 10.1371/journal.pone.0070344

**Published:** 2013-08-21

**Authors:** Hong-Ha M. Truong, Michael Grasso, Yea-Hung Chen, Timothy A. Kellogg, Tyler Robertson, Alberto Curotto, Wayne T. Steward, Willi McFarland

**Affiliations:** 1 Department of Medicine, University of California San Francisco, San Francisco, California, United States of America; 2 Gladstone Institute of Virology and Immunology, San Francisco, California, United States of America; 3 Department of Public Health, San Francisco, California, United States of America; Asociacion Civil Impacta Salud y Educacion, Peru

## Abstract

**Introduction:**

Respondent-driven sampling (RDS) offers a recruitment strategy for hard-to-reach populations. However, RDS faces logistical and theoretical challenges that threaten efficiency and validity in settings worldwide. We present innovative adaptations to conventional RDS to overcome barriers encountered in recruiting a large, representative sample of men who have sex with men (MSM) who travel internationally.

**Methods:**

Novel methodological adaptations for the “International Travel Research to Inform Prevention” or “I-TRIP” study were offering participants a choice between electronic and paper coupons referrals for recruitment and modifying the secondary incentives structure from small cash amounts to raffle entries for periodic large cash prize raffle drawings. Staged referral limit increases from 3 to 10 referrals and progressive addition of 70 seeds were also implemented.

**Results:**

There were 501 participants enrolled in up to 13 waves of growth. Among participants with a choice of referral methods, 81% selected electronic referrals. Of participants who were recruited electronically, 90% chose to remain with electronic referrals when it was their turn to recruit. The mean number of enrolled referrals was 0.91 for electronic referrals compared to 0.56 for paper coupons. Median referral lag time, i.e., the time interval between when recruiters were given their referrals and when a referred individual enrolled in the study, was 20 days (IQR 10–40) for electronic referrals, 20 days (IQR 8–58) for paper coupons, 20 days (IQR 10–41) for raffle entries and 33 days (IQR 16–148) for small cash incentives.

**Conclusions:**

The recruitment of MSM who travel internationally required maximizing known flexible tools of RDS while at the same time necessitating innovations to increase recruitment efficiency. Electronic referrals emerged as a major advantage in recruiting this hard-to-reach population who are of high socio-economic status, geographically diffuse and highly mobile. These enhancements may improve the performance of RDS in target populations with similar characteristics.

## Introduction

For research studies that entail the recruitment of a hard-to-reach target population, obtaining a meaningfully large and representative sample can be difficult. A methodology from the field of sociological research known as respondent-driven sampling (RDS) offers an approach through the use of chain referrals with statistical adjustments to approximate a probability basis [1]–[3]. RDS uses long-chain referral whereby members of the target population recruit other members, similar to snowball sampling.

The RDS recruitment process starts with purposeful selection of an initial set of participants, also referred to as “seeds”. The seeds cover the diversity of the target population with respect to factors describing social groupings, such as demographic characteristics and behaviors of interest. Ideally, they have large social networks from which to recruit other eligible participants. Peer-recruited participants who enroll in the study or “enrolled referrals” would in turn refer additional participants. These successive recruitment cycles or “waves” continue until sample stability and targeted sample size are reached. A sample that reaches equilibrium provides a basis for adjusting estimates to be representative of the entire target population. Relative network sizes are used to estimate differential recruitment probabilities. Recruitment linkages between participants are used to estimate design effects and their impact on standard errors. The ultimate goal of obtaining a sufficiently large and representative sample is achievable if the RDS study adheres to several underlying theoretical assumptions, including respondents report the size of their social network accurately, respondents recruit randomly from their social network and network connections are reciprocal [4].

Efficiency of accrual and guarantee of participant eligibility with peer-referral strategies are based on the rationale that individuals who are members of the social network will have better access to the target population than outsiders, e.g., study staff. Validity of the sample is based on correcting for different probabilities of inclusion by network size and adjusting for similarities between the recruiter and recruits. RDS has been used in public health research studies around the world. A systematic review of global RDS studies concluded this methodology can be successfully implemented to recruit populations at high risk for HIV for behavioral and biological surveys [5]. In the field of HIV research, the target population is often high-risk groups such as injection drug users, female sex workers and men who have sex with men (MSM) [6]–[8].

 However, RDS faces logistical and theoretical challenges that could threaten efficiency and validity in settings worldwide. In our study, “International Travel Research to Inform Prevention” or “I-TRIP”, we encountered barriers to using conventional RDS methodology. We found our target population of MSM who travel internationally presented an unusual challenge in the recruitment of a representative sample. The challenges revolved around two key components of conventional RDS: use of paper coupons for recruitment and the secondary incentives structure.

In a conventional RDS study, participants are asked to recruit others from their social network upon completion of the survey. Those willing to recruit are provided with coded, non-replicable paper coupons to give to potential participants. The coupons are designed to help track and verify the recruiter-recruit relationship for the purposes of data analysis and secondary incentives. However, the need to meet face-to-face to give the coupons was problematic for many of our participants, especially those who travel frequently and whose peers also travel often. Another central aspect of RDS is the dual incentive structure. Study participants receive a primary incentive for completing the survey or interview process and are offered a secondary incentive for referral of individuals who ultimately enroll in the study. Secondary incentives are generally considered a small token of appreciation for successful referrals. For our study population which tended to be relatively financially stable, the small cash amount initially offered as secondary incentives may have been insufficient motivation to recruit other participants [9]. Based on anecdotal feedback received from our study participants, the use of paper coupons and cash secondary incentive affected their ability to provide referrals successfully, as reflected by the slow progress in recruitment.

We recognized that for our study to be successful, adjustments to conventional RDS methodology were needed. To overcome logistical difficulties posed by paper coupons and lack of appeal of the secondary incentive structure, we developed innovative ways to enhance the recruitment methodology. One innovation was the use of electronic referrals as an alternative to conventional paper coupons. Another modification was revising the secondary incentives structure from small amounts of cash upon enrollment of referrals to periodic large cash prize raffles. We also used known flexibilities in RDS, such as higher numbers of peer referrals per participant and re-seeding when chain growth lagged. We present here our field experiences with these novel adaptations as an illustrative case study of how modifications to conventional RDS can improve its feasibility and enhance the inclusion of special, hard-to-reach populations in research.

## Methods

### Ethics Statement

The study received approval from the Institutional Review Board at the University of California, San Francisco. Written consent was received from participants who completed the survey.

### Original Study Design

MSM who were 18 years or older, resided in the San Francisco Bay Area and had traveled internationally in the past 12 months were eligible for the I-TRIP study. Participants completed a quantitative survey and were tested for HIV. After the survey process was completed, participants were received a detailed explanation of the peer-referral process.

Participants were asked to identify members of their social network who recently traveled internationally and might be interested in the study. The social network size questions included: 1) “Approximately how many gay or other men who have sex with men, who live in the San Francisco Bay Area, do you know by name?”; 2) “As far as you know, of these men, how many have traveled to an international destination in the past 12 months?”; and 3) “Of these men who traveled internationally, to how many would you be able to give a paper coupon referral to in the next 4–8 weeks?” Participants who expressed a willingness to participate in the peer-referral process were given paper coupons conventionally used in RDS studies. Each coupon was coded with a unique identifier to allow linkages between the recruiter and their referrals and was tracked internally using a recruitment database. The paper coupons contained the study telephone number to enable interested potential participants to contact the study team and find out additional information prior to making an interview appointment. At the start of the study, each participant was provided with 3 paper coupons. For each enrolled referral, participants received a $10 secondary incentive.

Prior to launching the study, a formative assessment was conducted to obtain feedback from members of the target population on the use of RDS for the study. We conducted 8 key informant interviews and 2 focus group discussions. Participants of the key informant interviews and focus groups thought that using peer-referral was an appropriate recruitment strategy and expressed confidence that recruiting three eligible study participants would be feasible. Results from the formative assessment indicated that conventional RDS methodology could be implemented successfully to recruit our target population. However, enrollment rates were much lower than expected in the first few months of the study roll-out. This observation suggested that the formative assessment participants overestimated the ability of their peers to implement the conventional RDS methodology.

### Conceptualization of the Revised Methodology

The concepts of electronic referrals and prize raffles as secondary incentives emerged from internal discussions among study team members of ways to enhance the recruitment process. The study team reflected on how we communicated with our own social networks. This introspection led to the realization that a vast majority of our personal communication occurred electronically. The use of emails to provide referrals electronically appeared to be a good fit for our target population. With regards to incentives, we realized from the onset of the study that conventional reimbursements might not be sufficient for our study population who are better off financially than the target populations of most other RDS studies. Therefore, we originally planned to hold prize raffles at the halfway mark (250 enrolled participants) and at the end of the study. This concept was vetted during the formative assessment. However, these raffles turned out to be too far removed in the future to sufficiently motivate participants to recruit. This realization led to the idea of recruitment raffles that would be held on a more frequent basis and closer in time to the participant's enrollment in the study.

### Electronic Referrals

The electronic referral method was developed as an alternative means for participants to recruit their peers. Within the first couple months after roll-out, the study team began receiving feedback about recruitment difficulties. Even though participants could identify members of their social network who were interested in the study, it was difficult at times to arrange a meeting with potential recruits to give them the paper coupons. Potential participants were geographically spread out across the greater San Francisco Bay Area and often were traveling themselves. Other problems included a recruiter or recruit losing the coupon, as well as a recruit forgetting to bring the coupon to the interview appointment, therefore not be able to link to their recruiter and needing to reschedule. Taken together, these issues indicated the paper coupon had become a barrier for recruitment and enrollment.

Electronic referrals were integrated into the protocol in the fourth month of the study. The recruiter was asked to identify and discuss the study with members of his social network, an approach similar to that used with paper coupons. If an individual expressed an interest in participating, the recruiter asked for permission to provide his contact information (e.g., email address, phone number) to the study team. The recruiter then sent an email to the study team with the individual's name and contact information. Information about the potential participant was entered into the study database and assigned a unique coded identifier, similar to the paper coupon process. The study team contacted the potential participant to provide more information, answer any questions and schedule an interview appointment if the individual was willing. All individuals contacted by the study team were asked to confirm that they had given the recruiter permission to share their contact information. Just as with paper coupons, each of the electronic referrals counted towards the participant's referral limit, regardless of whether the referred individual ultimately enrolled in the study.

After the survey administration process was completed, an explanation of the paper coupon and electronic referral process was provided. Participants who were willing to engage in the peer-referral process were asked whether they preferred to recruit using paper coupons or electronic referrals. Participants were then asked social network size questions tailored to the referral method of choice. For example, participants who chose the electronic method were asked, “Of these men who traveled internationally, to how many would you be able to give an electronic referral in the next 4–8 weeks?” Irrespective of the recruitment methodology they chose, participants were offered the same number of referrals to use for recruitment and provided with both a paper and an electronic version of an information sheet to assist them with discussing the study with potential recruits.

### Prize Raffles as Secondary Incentives

In the first three months into the study, there was a low rate of referrals. Of the first 13 participants who provided referrals that went on to enroll in the study, only 2 individuals collected their secondary incentives. This observation suggested that the current secondary incentive was not sufficient to motivate participants to recruit others and likely was contributing to the low number of enrolled referrals.

The secondary incentives structure was revised in the fourth month of the study. In place of the conventional offering of a small cash amount ($10), participants received one raffle entry for each enrolled referral. After every fiftieth enrolled referral, a recruitment raffle was held for a $500 gift card prize.

### Follow-Up Survey

Participants who went on another international trip within 12 months following their baseline survey were eligible to complete a follow-up quantitative survey. A component of the follow-up survey was a series of open-ended questions asking participants about their recruitment experiences. Participants were asked to describe successes and challenges to recruiting from their social network. Participants who recruited others using electronic referrals were asked the reasons why they chose electronic referrals and whether they would choose this referral methodology again in the future.

## Results

A total of 501 participants were enrolled in the I-TRIP study. Demographic characteristics of the study population and crude and RDS-adjusted percentages are presented in Table 1. Participants were mostly white (n = 326), residents of San Francisco (n = 425), self-identified as homosexual or gay (n = 479), HIV-negative (n = 370) and had a college degree or higher (n = 418). The median age was 40 years old.

**Table 1:** Participant demographic characteristics for the I-TRIP Study (N = 501).

	Number	Crude %	Adjusted %	95% CI
**Age**				
18–25	38	7.6	11.3	5.0, 16.5
26–30	85	17.0	16.9	11.7, 24.5
31–35	62	12.4	10.7	6.5, 15.9
36–40	71	14.2	14.1	9.5, 20.6
41–45	83	16.6	15.0	9.8, 19.4
46–50	52	10.4	11.4	6.4, 15.5
≥51	110	21.9	20.6	14.3, 29.6
**Race/Ethnicity**				
White	326	65.0	58.8	49.7, 65.9
Asian/Pacific Islander	75	15.0	19.4	12.6, 29.0
Hispanic/Latino	68	13.6	13.9	8.8, 18.8
Black	12	2.4	2.5	0.1, 5.6
Other/Mixed	20	4.0	5.4	2.4, 9.1
**Sexual Orientation**				
Homosexual/Gay	479	95.6	91.9	84.6, 96.6
Bisexual	19	3.8	7.1	2.6, 14.4
Heterosexual/ Something else	3	0.6	1.0	0.0, 2.3
**Highest Education Level**				
High school/GED	18	3.6	5.2	2.1, 8.8
Some college	65	13.0	17.9	12.9, 26.4
College degree	234	46.7	44.2	36.6, 50.5
Graduate school	184	36.7	32.7	25.0, 39.0
**County of Residence**				
Alameda	47	9.4	13.3	7.4, 19.6
Contra Costa	6	1.2	2.7	0.3, 5.4
Marin	5	1.0	1.0	0.1, 2.0
Napa	0	0	0	0
Santa Clara	4	0.8	0.7	0.0, 2.4
Santa Cruz	0	0	0	0
Sonoma	3	0.6	1.7	0.0, 4.4
San Francisco	425	84.8	77.7	70.4, 86.4
Solano	1	0.2	0.1	–, –
San Mateo	10	2.0	2.8	0.3, 4.6
**HIV test results**				
Negative	370	73.9	69.0	60.0, 77.0
Positive	128	25.5	28.6	21.5, 38.7
Indeterminate	3	0.06	2.4	0.0, 4.1

**Figure 1:**
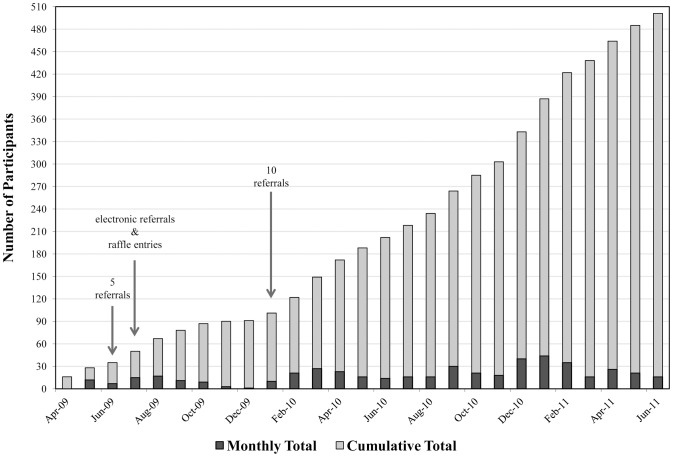
Timeline of monthly and cumulative participant enrollment and implementation of revised recruitment methodologies for the I-TRIP Study (N = 501).

Study participants were enrolled over a 27-month period from April 2009 through June 2011, as shown in Figure 1. Targeted sample size was reached, although accrual was slow overall. There were 149 participants enrolled during year 1 of the study, at an average rate of 12 participants per month. During year 2, there were 289 participants enrolled at an average rate of 24 participants per month, which was double the enrollment of the previous 12 months. There were 63 participants enrolled during the final 3 months of the study at an average rate of 21 participants per month, which was similar to year 2.

**Figure 2:**
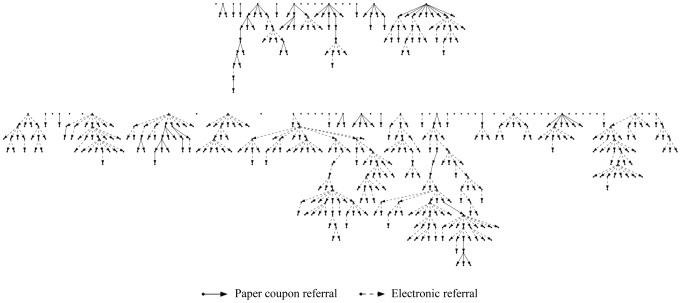
I-TRIP study network profile (N = 501) of recruiters and enrolled referrals. Seeds represent the first row of each image and are presented in the order of enrollment. Seeds offered only paper coupon referrals are shown in the top image; seeds with a choice between paper coupons and electronic referrals are shown in the bottom image.

A study network profile of I-TRIP study participants was generated by linking recruiters and their enrolled referrals through their unique identifiers, as presented in Figure 2. Recruitment chains ranged from 2 generations (seed and one wave of referrals) up to 13 generations (seed and 12 waves of referrals). The distribution of participants by wave generations were as follows: 70 seeds (14.0%), 96 second generations (19.2%), 96 third generations (19.2%), 83 fourth generations (16.6%), 32 fifth generations (6.4%), 23 sixth generations (4.6%), 17 seventh generations (3.4%), 21 eighth generations (4.2%), 26 ninth generations (5.2%), 17 tenth generations (3.4%), 15 eleventh generations (3.0%), 2 twelfth generations (0.4%) and 3 thirteenth generations (0.6%). The median number of wave generations was four.

Equilibrium was reached by the 5^th^ wave generation for all demographic characteristics, including age, race/ethnicity, sexual orientation and education level. An assessment of the recruiter-recruitee relationship indicated that participants referred a variety of individuals from their social network, including friends (61%), co-workers (10%) and husband/domestic partner/boyfriend/sexual partner (9%). Homophily was assessed on a scale of −1 (participant recruited only from outside his group) to 1 (participant recruited only from inside his group). Values at either extreme of the homophily scale were rarely observed among the variables of age, race, sexual identity, education level, county of residence and HIV status.

Out of the 70 seeds, 41 provided at least one enrolled referral and 39 did not provide any enrolled referrals. There were 11 seeds enrolled in the first 2 weeks of the study. An additional 10 seeds where enrolled in the following 6 months to replace the unproductive seeds. Throughout the study, referral patterns and enrollment rates were monitored closely. During periods when enrollment slowed down considerably and referral chains appeared to be dying out, additional seeds were added in order to refresh the referrals process.

The first 35 participants enrolled were only offered paper coupons. The last 2 participants enrolled were not offered the option to recruit additional participants because the study was nearing completion. Of the 464 participants presented with a choice between paper coupons and electronic referrals, 378 participants (81.5%) selected electronic referrals, 85 (18.3%) chose paper coupons and 1 participant (0.2%) declined to provide referrals. There were 340 participants recruited by the electronic method who were offered and accepted the option to recruit additional participants. Among those 340 participants, 306 (90%) subsequently chose electronic referrals and 34 (10%) chose paper referrals. Of the 71 participants recruited by the paper coupon method, 37 (52%) subsequently chose electronic referrals and 34 (48%) chose paper referrals. The mean number of enrolled referrals was 0.91 for the electronic method compared to 0.56 for the paper coupons. Median referral lag time, i.e., the time interval between when the recruiters were given their referrals and when a referred individual enrolled in the study, was 20 days (IQR 10–40) for participants using electronic referrals and also 20 days (IQR 8–58) for those using paper coupons.

The final modification to the recruitment process was a staged increase of referral limits. The referral limit increased from 3 to 5 in the fourth month of the study and to 10 in the tenth month. Of the 395 participants who were offered 10 referrals and a choice of recruitment methods, 335 (84.8%) chose electronic referrals and 60 (15.2%) chose paper coupons. The return rate, i.e., the proportion of referrals that resulted in enrolled participants, was 8.4% for electronic referrals and 6.3% for paper coupons. When non-productive seeds were excluded (n = 30), the return rate was 8.9% for participants using electronic referrals (n = 314) and 7.5% for those using paper coupons (n = 51).

A subset of 52 participants who were offered a choice of referral methods participated in a follow-up survey component of the study. Among these individuals, 33 had chosen electronic referrals and 19 had chosen paper coupons. Participants were asked about their experiences with the referral process. The main reason given for choosing electronic referrals over paper coupons was that they thought it would be easier to provide referrals without having to meet in-person to conduct the coupon exchange (86%). The most common methods used by participants to inform their social network about the study included forwarding the study email that they received which contained instructions on the referral process (42%), speaking with them over the phone (30%) and writing an email describing the study (21%). All participants who used electronic referrals indicated that they would choose this method again due to the convenience of the referral process. Participants commented that they believed the electronic referrals enabled them to contact a larger proportion of their social network in a more expedient fashion.

The first 35 participants enrolled were offered the $10 cash incentive for each enrolled referral while the next 464 participants were offered one raffle entry for each enrolled referral. Median referral lag time was 33 days (IQR 16–148) among participants who were offered the $10 cash incentives and 20 days (IQR 10–41) among those offered raffle entries.

## Discussion

Our study on HIV risk and international travel presented the challenge of recruiting a hard-to-reach MSM population. The modified approach of electronic referrals and prize raffles was able to achieve a large and diverse sample size. Accrual was slower than hoped for and expected in typical RDS studies, most likely due to the enrollment criteria of recent international travel which considerably narrowed the pool of eligible potential participants. The electronic referral method emerged as a clear advantage over conventional RDS paper referral coupons, having been selected by more than a three to one margin by the I-TRIP study participants who were given a choice between the two recruitment methods. There was very little cross-over of referral methods among participants who were recruited electronically, as the overwhelming majority chose to remain with electronic referrals when it came their turn to recruit. The convenience of contacting potential recruits was cited as the primary benefit of the electronic referral method.

Out of necessity, we leveraged and innovated upon conventional recruitment methods of RDS while attempting to adhere to underlying theoretical assumptions. The assumption of random selection of peers within the social network would likely be comparable whether the participant chose electronic or paper coupon referrals, since the initial conversation with potential participants about the study could take place by phone, email, text messages or in person, irrespective of referral method. The primary difference with electronic referrals is that participants would not have to arrange in-person meetings to transfer the coupon. Electronic referrals, therefore, might actually enhance random selection of peers since participants would not need to limit their potential referrals to only those individuals whom they would be able to meet with in person.

The innovation of having participants provide their referrals via email takes advantage of the fact that the Internet has become a large component of everyday life. A recent survey found 85% of adults in the United States used the Internet and that 91% of them used the Internet to send or read emails [10]–[11]. Internet use was highest among 18–29 year olds (96%), 30–49 year olds (93%), those with some college (94%) and those with a college or higher degree (97%), the same demographic characteristics that represent the majority of our study participants. Provision of referrals through text messaging is a potential variation of the electronic referral process and may be more suitable in settings where email use is not as ubiquitous.

A revised incentive structure also enhanced recruitment over what was achieved with conventional secondary incentives. Participants offered raffle entries provided enrolled referrals more quickly than those who were offered the $10 cash as a secondary incentive. Receipt of raffle entries for an opportunity to win a $500 gift card appeared to be more appealing to participants than receiving the guaranteed $10 per enrolled referral. Prior to the modification, only 15% of participants collected their cash incentive. This finding is comparable to figures in the 20% range that are cited by other RDS studies [12].

Other studies have used Web-based variations of RDS. A study of alcohol and drug use among young adults allowed participants to use social media to forward the link for their unique identifying number [13]. This referral method inadvertently permitted multiple individuals to begin a survey at the same time and led to referral limits being exceeded. In contrast, our electronic referral method was designed to enable the study team to monitor the number of referrals as they were being submitted and thereby curtail additional referrals once the limit had been reached. Another Web-based study sent the participants a set number of recruitment emails with unique serial numbers and asked them to forward each of the emails to one potential recruit [12]. The necessity for potential recruits to have an email address in order to participate and the possibility of duplication since one individual could have multiple email addresses were limitations in this study that were not encountered in the I-TRIP study using our electronic referral method.

Use of electronic referrals and raffle prizes resulted in an overall cost savings to the study, primarily due to the fact that they yielded shorter referral lag times compared to paper coupons and small cash incentives. More rapid accrual of participants helped to shorten the study period, which translated to reduced study personnel costs and limited the potential noise from temporal events. Had the original study design of paper coupons and small cash incentives been retained, it very likely would have taken more than 2 years to enroll the targeted sample size of 500 participants. The $500 prize for the recruitment raffle held after every fiftieth enrolled referral was equivalent to the cost of paying $10 cash for each of those 50 enrolled referrals. It is possible that retaining the $10 cash incentive may have saved money if a low proportion of participants collected their secondary incentives. However, slower accrual of participants would have lengthened the time it took to recruit the sample, thereby resulting in increased personnel costs and effectively negate any cost savings from the uncollected secondary incentives. Electronic referrals also saved on the expense of printing paper coupons.

A number of methodological adjustments to the referral methodology and secondary incentive structure were implemented at various stages in the study. Overlapping implementation of these modifications made it is difficult to pinpoint which procedural revision had the greatest impact on increasing recruitment numbers. The primary goal of these ad-hoc modifications was to enhance study enrollment, as this study was not designed to be a randomized controlled trial to compare referral methods and incentive structures.

Electronic referrals were clearly the preferred recruitment method, a conclusion supported by the large proportion of participants who chose this option over paper coupons. Electronic referrals yielded better recruitment results than paper coupons, as evidenced by the greater number of referrals that were provided at a faster rate. More rapid recruitment is most likely attributable to the fact that the electronic method enabled the study team to be more proactive upon receipt of referrals. Having the contact information of referrals enabled the study team to reach out actively to potential participants rather than wait passively for participants to contact the study. The study team could then provide potential participants with additional information and answer questions about the study. Enrolled participants were given the option to choose the recruitment method they thought would be more successful. While this procedure may have introduced self-selection bias, the implementation of electronic referrals was designed primarily to increase enrollment and not as a comparison of referral methods. Future research on the use of electronic referrals can control for potential sampling bias by randomizing participants by referral methods.

The recruitment of MSM who travel internationally required maximizing known flexible tools of RDS while at the same time necessitating innovations to increase recruitment efficiency. Our experience highlights the importance of designing a study that is tailored to reach the population of interest and includes feedback loops to provide information that can assist in pinpointing problems that arise during implementation. Electronic referrals emerged as a major advantage in recruiting this hard-to-reach population who are of high socio-economic status, geographically diffuse and highly mobile. These enhancements may improve the performance of RDS in target populations with similar characteristics.
